# Antibiotic prescription in the treatment of odontogenic infection by health professionals: A factor to consensus

**DOI:** 10.4317/medoral.17504

**Published:** 2011-12-06

**Authors:** Raquel González-Martínez, Isidoro Cortell-Ballester, José M. Herráez-Vilas, José M. Arnau-de Bolós, Cosme Gay-Escoda

**Affiliations:** 1 DDS. Resident of the Master of Oral Surgery and Implantology. University of Barcelona Dental School; 2 DDS. Master in Oral Surgery and Implantology. University of Barcelona Dental School; 3 DDS. Master in Oral Surgery and Implantology. Professor of the Master of Oral Surgery and Implantology. University of Barcelona Dental School. IDIBELL Research group; 4 MD, PhD. Professor of Pharmacology, Faculty of Medicine, University of Barcelona. Head of Clinical Pharmacology University Hospital of Bellvitge. IDIBELL Research group; 5 MD, DDS, PhD. Chairman of Oral and Maxillofacial Surgery. Director of the Master in Oral Surgery and Implantology. University of Barcelona Dental School. Oral and maxillofacial surgeon of the Teknon Medical Center, Barcelona (Spain). IDIBELL Research group

## Abstract

Objective: To observe the attitude of dentists and family doctors in prescribing antibiotics for the treatment of dental infections. Study Design: A poll was performed to determine the differences in the prescription of antibiotics for the treatment of odontogenic infection by dentists and family doctors of the primary care department of the Catalan Health Care Service. Results: A hundred polls were distributed among family doctors, and another 100 ones among primary care dentists assigned to the Catalan Health Care Service of the Generalitat de Catalunya. Of the total of questionnaires distributed, 63 were retuned and answered from dentists and 71 from family doctors. Eighty-one percent of dentists included in the opinion poll considered amoxicillin as the first antibiotic choice for the treatment of odontogenic infections, while 73.2% of family doctors preferred the combination of amoxicillin and clavulanic acid. With regard to antibiotics of choice in patients allergic to penicillin, 67.7% of family doctors preferred macrolides (25.4% opted for clarithromycin, 25.4% for erythromycin and 16.9% for spiramycin). However, clindamycin was the antibiotic most frequently prescribed by dentists (66.7%), followed by erythromycin (28.6%). Conclusions: The results of this study show a large discrepancy in the criteria for the treatment of odontogenic infections on the part of leading professionals involved in the management of this condition. Although the most common prescription involved beta-lactam antibiotics in both groups, several significant differences have been detected with regard to the second antibiotic choice.

** Key words:**Odontogenic infections, antibiotics, antimicrobials.

## Introduction

Although the incidence of odontogenic infections has declined in recent years as a result of the improvement in oral health care, it is still the main cause of infectious disease in Spain and the most common reason for consultation and intervention by dentists (1). Odontogenic infections affect the entire population, from children to the elderly. The most frequent are those resulting from dental caries, dentoalveolar infections (infections of the pulp and periapical abscess), gingivitis, periodontitis (including peri-implantitis), aponeurotic space infections, osteitis and osteomyelitis (2,3).

It is estimated that in Spain odontogenic infections represent around 12% of antibiotic prescriptions. These prescriptions are dispensed by dentists in 62% of cases and by family doctors in 36% of other cases (3).

The treatment of odontogenic infections is based on two main elements: surgical treatment and antibiotic therapy. In most cases antibiotic prescription is empirical and relates to a number of factors that are not always well known and defined, and among which are included the perception efficacy, the knowledge of empirical therapy recommendations, opinions regarding the most common etiology, the expected bacterial resistance as well as knowing the existence of different antibiotics. Other aspect are also relevant, such as the presence of allergies, comorbidity, the prospect of receiving antibiotics, previous experiences and the trust between health professionals and patients. As a result, treatments may often be inappropriate and promote the development of bacterial resistance (1,4 to 6).

Several studies have been conducted in Spain in the past 10 years that describe the behavior of health professionals in primary care with regard to prescribing antibiotics; these studies show that there is an excessive prescription of antibiotics, as well as an inappropriate use of them (7). The objectives of our study are to assess the performance of dentists and family doctors in prescribing antibiotics for the treatment of odontogenic infections, as well as determine the differences between both groups.

## Material and Methods

A cross-sectional study was carried out by distributing 200 polls among dentists and family doctors of the primary care department of the Catalan Health Service (Generalitat de Catalunya, Spain) to determine the differences in prescribing antibiotics for the treatment of odontogenic infection. Professionals were asked whether they regarded the antibiotic treatment as the first and second choice, and whether they regarded penicillin as the treatment of choice in penicillin allergy patients. The poll consisted of 3 questions: 1) In patients with odontogenic infections, what antibiotic would you prescribe as first choice? 2) In patients with odontogenic infections, what antibiotic would you prescribe as second choice? and 3) In patients with odontogenic infections and allergic to penicillin, what antibiotic would you prescribe?. The poll was anonymous, the only personal information requested included age and years of practice.

Data were analyzed using the Statistical Package for Social Science version 15.0 for Windows (SPSS inc., Chicago, IL, USA. License granted to the University of Barcelona). The descriptive analysis of the data was carried out by the distribution of frequencies.

## Results

**Figure 1:**
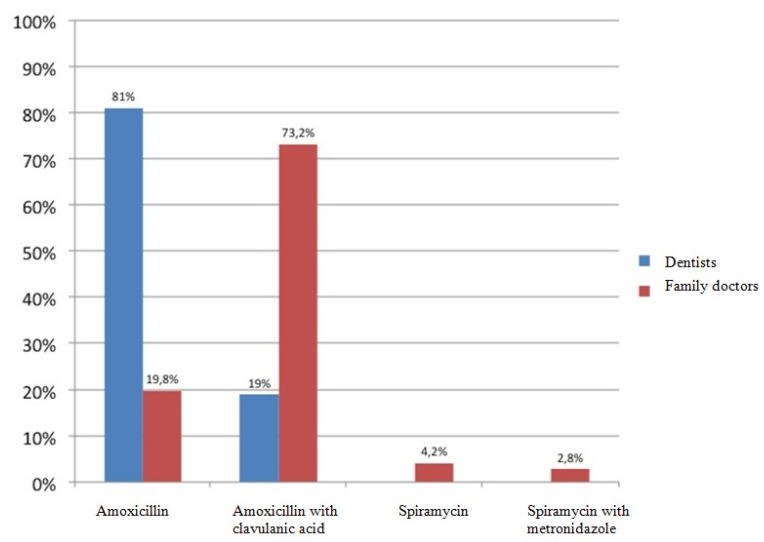
First-choice antibiotics prescribed by dentists and family doctors.

**Figure 2:**
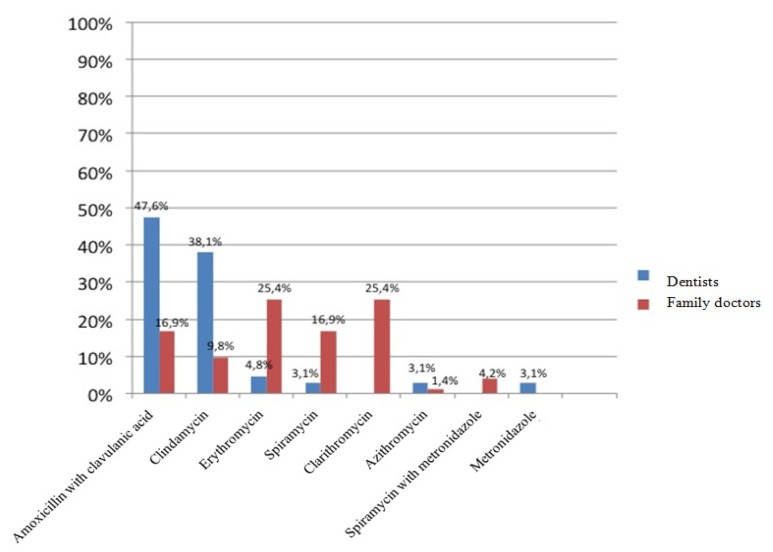
Second-choice antibiotic prescribed by dentists and family doctors.

**Figure 3:**
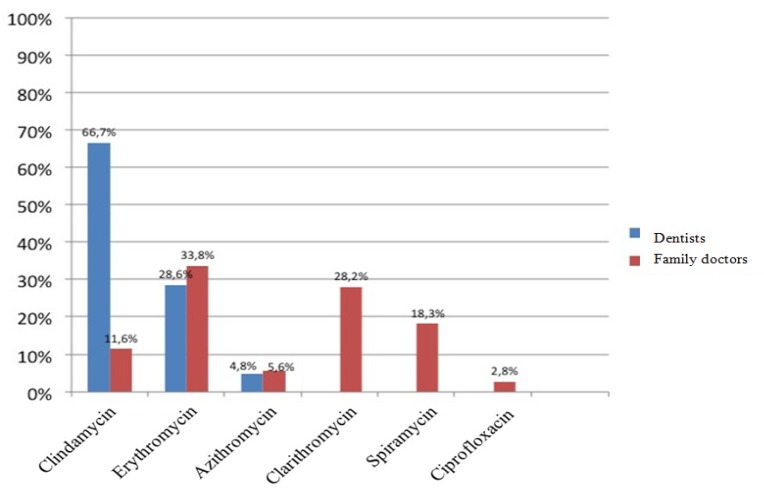
Antibiotics of choice in penicillin-allergic patients prescribed by dentists and family doctors.

A hundred polls were distributed among family doctors and another 100 among dentists of the primary care department of the Catalan Health Service. Of all questionnaires distributed, 63 were returned and answered by dentists and 71 by general practitioners. The average time of professional practice of dentists was 10.5 years, with a range between 1 and 20 years, while the average time referred by family doctors was 20.94, with a range between 3 and 40 years. Eighty-one percent of dentists surveyed considered amoxicillin the first antibiotic choice for the treatment of odontogenic infections, while 73.2% of family doctors preferred the combination of amoxicillin and clavulanic acid (Fig. 1). With respect to the second antibiotic choice, 67.7% of family doctors opted for macrolides - 25.4% opted for clarithromycin, 25.4% for erythromycin and 16.9% for spiramycin -. However, among dentists the second antibiotic choice most often prescribed was amoxicillin in combination with clavulanic acid (47.6%), followed by clindamycin (38.1%) (Fig. 2). In patients allergic to penicillin, clindamycin was the antibiotic most often prescribed by dentists (66.7%) followed by erythromycin (28.6%), while among family doctors, the most frequently prescribed antibiotic was erythromycin (33.8%), followed by clarithromycin (28.2%). Only 11.6% of family doctors prescribe clindamycin to penicillin-allergic patients (Fig. 3).

## Discussion

In recent years, some authors have warned about the high use of antibiotics in Spain. This fact is not justified by a higher prevalence of infections in this country when compared with others. However, there is a tendency to prescribe antibiotics for any infection, regardless of its etiology. Although it requires a formal prescription from a practitioner (a doctor or a dentist) to buy antibiotics, 30% of all the sales of antibiotics in Spain are done without this requirement (8-10). Moreover, the rapid introduction of new antimicrobial molecules has helped to increase the rate of resistance, which is one of the highest in Europe (11).

In our study the most frequent prescription referred by both groups of respondents for the treatment of odontogenic infection was betalactams. However, most family doctors prescribe amoxicillin in combination with clavulanic acid, while dentists only prescribe amoxicillin. These data are consistent with those provided by Hail et al. (3) who claim that 71% of dentist’s prescriptions are beta-lactam antibiotics, with 48% of prescriptions for amoxicillin and 23% to amoxicillin in combination with clavulanic acid. On the other hand, 59% of family doctors’ prescriptions are beta-lactams, but 41% of them in combination with clavulanic acid, and 16% are amoxicillin prescriptions.

The most important limitation of this study is the low number of questionnaires analyzed; a fact that makes difficult to extrapolate the results to all the Spanish primary care dentists and family doctors. However, in our opinion, these results may help understand the current situation for prescribing antibiotics for the treatment of odontogenic infections by health professionals involved in the management of such drugs.

Another limitation is that questionnaires should be simple and brief, as well as quick and easy to read and complete. This means the omission of relevant information that the clinician should consider before deciding what antibiotics should be prescribed.

Anyway, it is noteworthy that 3% of family doctors prescribe spiramycin, with or without metronidazole, as the first antibiotic choice for the treatment of odontogenic infections. This data contrast with the recommendations made by most authors, such as Bresco-Salinas et al. (1), who recommend the use of amoxicillin as the first drug choice or Bascones et al. (12) and Isla et al. (13) who recommend amoxicillin in combination with clavulanic acid. These authors analyzed the efficacy of antibiotics commonly used in dentistry to destroy the most common bacteria in odontogenic infections. They performed pharmacokinetic and pharmacodynamic analysis and concluded that amoxicillin in combination with clavulanic acid, and clindamycin are adequately effective against microorganisms isolated from odontogenic infections, while spiramycin and metronidazole do not cover the bacterial spectrum bacterial of this type of infections (13).

The number of microorganisms that have become resistant to common antibiotics used in the treatment of odontogenic infections has doubled in the last 15 years (1,14 to 20). In this context, Herrera et al. (14) found beta-lactamase-producing species in 87.1% of patients with periodontitis. Similarly, Bresco-Salinas et al. (1) observed high resistance to metronidazole (50.5%), erythromycin (39.1%) and azithromycin (33.2%) in odontogenic infections caused by bacteria.

Sobottka et al. (19) isolated 87 pathogens from 37 patients with periapical abscesses; they found that 100% of the pathogens were susceptible to amoxicillin in association with clavulanic acid, but sensitivity decreased between 70% and 75% for doxycycline, clindamycin and amoxicillin.

Some authors (19,20) recommend the use of moxifloxacin as the second antibiotic choice. Sobottka et al. (19) found 60% of strains resistant to erythromycin and Thomas et al. (20) found 12% of strains resistant to clindamycin. However, with regard to sensitivity of pathogens to quinolones, Thomas et al. (20) observed 100% of strains susceptible to moxifloxacin, whereas Sobottka et al. (19) found 98% of strains susceptible to moxifloxacin and levofloxacin.

However, other authors (21) affirm that the introduction of moxifloxacin for the treatment of odontogenic infections can contribute to an alarming increase in fluoroquinolone resistance.

In conclusion, we can say that there is a clear discrepancy among family doctors and dentists in the prescription of antibiotics for the treatment of odontogenic infections. Beta-lactams are the first antibiotics of choice, while clindamicine is the substitute for penicillin-allergic patients. On the other hand, we believe that it is not justified the prescription of macrolides as the first antibiotic choice in the treatment of odontogenic infections. Finally, in our opinion it would be a good initiative to develop a clinical practice guideline to unify criteria and achieve maximum effectiveness in the treatment of odontogenic infections.
